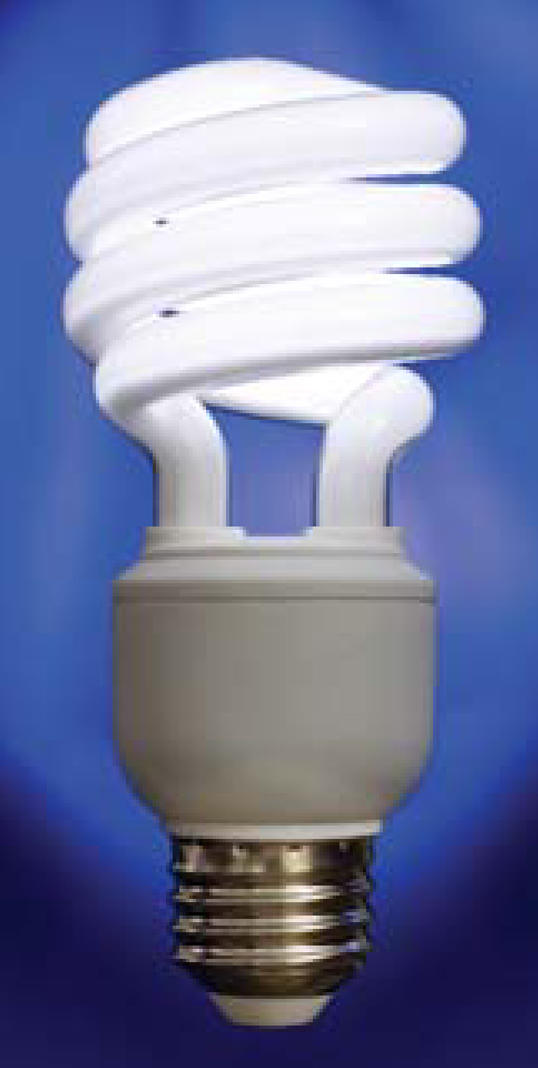# The Beat

**Published:** 2007-06

**Authors:** Erin E. Dooley

## Fresh Guidance for Produce Safety

Between 1998 and 2004, the number of produce-related disease outbreaks in the United States doubled, and outbreaks in the past six months have raised awareness more than ever. Now the FDA has called on industry to voluntarily implement the Hazard Analysis and Critical Control Point (HACCP) system throughout supply chains for fresh-cut produce such as shredded lettuce, bagged spinach, and peeled baby carrots. Some consumer groups have argued that HACCP should be mandatory for produce (as it is for meat), but the FDA counters that voluntary measures can be implemented more quickly than mandatory laws and may eventually lead to stricter controls.

## Water Prices Surge

Over the past five years, municipal water rates have risen by an average of 27% in the United States, 45% in Australia, 50% in South Africa, and 58% in Canada, according to an Earth Policy Institute report released in March 2007. Yet these price hikes do not affect developed countries only. In Tunisia, for example, the price for irrigation water has increased fourfold over the last decade. The report states that three main factors influence water prices: cost of transporting water from source to consumer, total demand, and price subsidies. The Earth Policy Institute advocates pricing water based on its true value and scarcity, a move the group feels will promote conservation.

## White House, Greenhouse

The Supreme Court’s 2 April 2007 ruling that greenhouse gases are pollutants does not require the U.S. EPA to regulate the gases, but does direct the agency to study the gases anew and regulate them if they are proven to harm human health. President Bush has said he plans no caps on greenhouse gases, and on 14 May 2007 reiterated his proposal to depend on a fivefold increase in cleaner fuels by 2017 to reduce emissions. The next day, the Senate defeated a proposal requiring the Army Corps of Engineers to consider climate change when designing water resources projects. Proposal sponsor John Kerry (D–MA) was not deterred, saying, “We’re making a statement here in the Senate to finally, once and for all, recognize the reality of what is happening with respect to climate change.”

## National Children’s Study Receives More Funding

In February 2007 Congress earmarked $69 million for the National Children’s Study. These funds will expand the study by 15 to 20 new study centers and will allow the 7 existing centers to begin recruitment. The study, under the auspices of a coalition of federal agencies, will eventually include 100,000 children from a total of 105 communities across the United States, following the children from before birth until age 21. Study researchers will focus on a wide range of health issues including birth defects, asthma, obesity, behavior, and environmental and dietary impacts on health. Findings should guide new disease prevention strategies and health and safety measures, as well as provide the impetus for potential disease treatments and cures.

## Urban Environment Report

The Earth Day Network released its first *Urban Environment Report* in February 2007, ranking the 72 largest cities in the United States by more than 200 environmental, health, and quality of life indicators. The indicators cover eight main subject areas including vulnerable populations, toxics and waste, air quality, drinking and surface water, human and public health, and climate change. The five cities ranking the lowest are Detroit, Miami, Cleveland, El Paso, and Houston, while the five highest-ranking cities are Fargo, Burlington (Vermont), Portland (Oregon), Colorado Springs, and Sioux Falls. The full report is available at http://www.earthday.net/UER/report/.

## The Great Lightbulb Switch

A group of industry players, environmental advocates, and energy specialists is working to phase out incandescent lightbulbs by 2016 in favor of new technologies including compact fluorescent lamps (CFLs) and light-emitting diodes, which are up to six times as efficient as incandescents and last much longer. About 150 million CFLs were sold in 2006. A complete changeover could save $18 billion in electricity annually and save the amount of power generated by as much as 80 coal-fired plants’ worth. What’s the rub? CFLs contain an average of 5 mg of mercury each. On the bright side, some retailers, such as IKEA, already have takeback programs in place, and advocacy groups are urging big retailers such as Wal-Mart to join the effort.

## Figures and Tables

**Figure:**
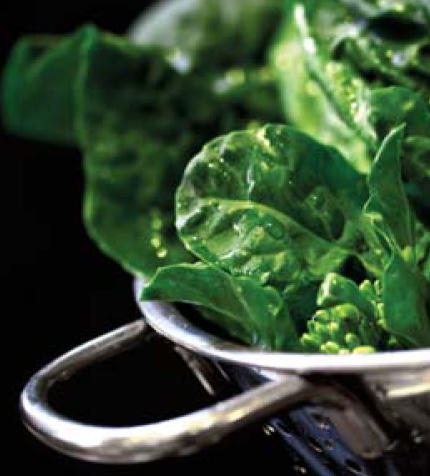


**Figure:**
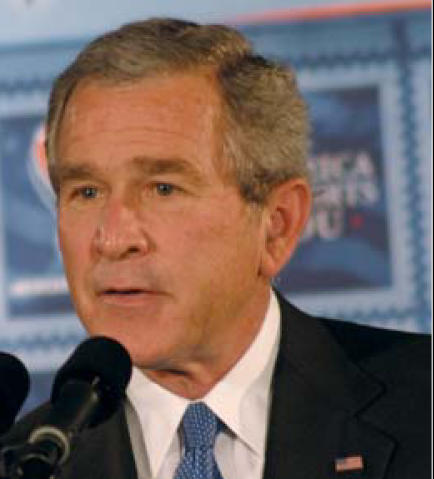


**Figure:**
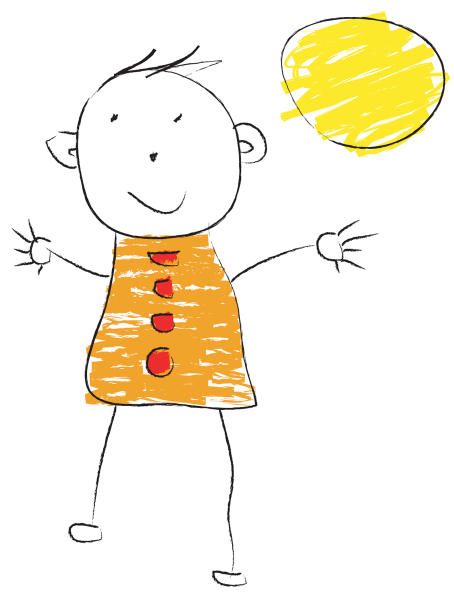


**Figure:**